# Relation of chair rising ability to activities of daily living and physical activity in Parkinson’s disease

**DOI:** 10.1186/s40945-020-00094-8

**Published:** 2020-12-08

**Authors:** Mon S. Bryant, Gu Eon Kang, Elizabeth J. Protas

**Affiliations:** 1 Medical Care Line, Michael E DeBakey Veterans Affairs Medical Center, 2002 Holcombe Blvd (Mail code 153), Houston, TX 77030 USA; 2 Department of Physical Medicine and Rehabilitation, Baylor College of Medicine, Houston, TX USA; 3 Interdisciplinary Consortium on Advanced Motion Performance (iCAMP), Baylor College of Medicine, Houston, TX USA; 4 School of Health Professions, University of Texas Medical Branch, Galveston, TX USA

**Keywords:** Activities of daily living, Chair rise, Parkinson’s disease, Physical activity

## Abstract

**Background:**

Many persons living with Parkinson’s disease (PD) have difficulty rising from a chair. Impaired ability to perform the chair rise may be associated with low physical activity levels and reduced ability to perform activities of daily living (ADL).

**Methods:**

Cross-sectional analysis was performed in 88 persons with PD to study the association of chair rising ability with ADL and physical activity.

**Results:**

We found that the participants who pushed themselves up from the chair had more severe PD, higher motor impairment and more comorbidity than those who rose from a chair normally. The Unified Parkinson’s Disease Rating Scale ADL (UPDRS-ADL), Schwab and England Activities of Daily Living Scale (SE-ADL) and the Physical Activity Scale for the Elderly (PASE) scores for the participants who pushed themselves up to rise (17.20 ± 7.53; 76.67 ± 13.23; 46.18 ± 52.64, respectively) were significantly poorer than for those who rose normally (10.35 ± 3.79; 87.64 ± 8.30; 112.90 ± 61.40, respectively) (all *p* < .05). Additionally, PASE scores were significantly poorer for participants who pushed themselves up to rise compared to those who rose slowly (95.21 ± 60.27) (*p* < .01). Pushing up to rise from a chair was a significant predictor of UPDRS-ADL (β = .357; *p* < .001; R^2^ = .403), SE-ADL (β = −.266; *p* = .009; R^2^ = .257) and PASE (β = −.250; *p* = .016; R^2^ = .162).

**Conclusions:**

Ability to rise from a chair was associated with ADL limitation and physical activity in persons with PD. Poor ability to rise from a chair may prevent persons from living independently and engaging in physical activity.

## Introduction

Rising from a chair, bed or toilet seat is a physically demanding function required to maintain independent living [1]. Many persons living with Parkinson’s disease (PD) have difficulties in rising from a chair, especially as PD progresses. For example, in a survey study of 101 persons with PD, 81% of respondents reported having difficulty with rising from a chair [2].

While motor planning, sensory/proprioceptive input and balance are critical for completing a rising from a chair task, the task also requires hip and leg extension strength [3, 4]. However, many older adults and persons with PD have insufficient leg strength to successfully perform the task quickly and to prevent falling back into the chair [3, 4]. Because rising from a chair requires greater leg-extensor strength and joint range of motion than other activities of daily living (ADLs), such as walking or stair climbing [5], difficulties in this ADL can lead to a sedentary lifestyle and a subsequent deterioration in overall daily function [6].

Evidence suggests an association between leg strength and overall daily function. For example, in stroke survivors, decreased lower-limb extensor power was associated with increased global indices of activity limitation [7]. Furthermore, reduction in leg strength has been considered the leading factor in functional deterioration in daily activities in older adults, specifically in such ADLs as standing up from a seated position, walking or climbing stairs [8].

Despite the presumption that poor chair rising ability can negatively influence both overall physical activity and ADL performance, the association between rising from a chair, physical activity and ADL has never been reported in PD. Therefore, in this study, we aimed to investigate the association of chair rise ability, physical activity and ADL in community-dwelling persons with PD. We expect that chair rising ability is a proxy measure of lower limb strength, and therefore our primary hypothesis was that impaired ability to perform the chair rise would differentiate physical activity levels and reduced ability to perform ADLs. Our secondary hypothesis was that worse chair rise performance would be selected from all measured variables as predictive of reduced ADL and physical activity levels in community-dwelling persons with PD.

## Materials and methods

### Participants

We performed a secondary analysis of a subset of a dataset which was published elsewhere [9]. Eighty-eight persons with PD were studied after they agreed to participate in the study and signed the informed consent form that was approved by the Institutional Review Board for Human Subject Research of Baylor College of Medicine (#20825) and the University of Texas Medical Branch (Human Research Ethics Committee [HREC] project reference number #06158).

### Inclusion/exclusion criteria

Participants were persons who had a diagnosis of idiopathic PD confirmed by a movement disorder specialist. Persons who had a diagnosis of atypical Parkinsonism, or had stereotactic surgery or deep brain stimulation for PD were excluded. All participants were recruited from movement disorder clinics at hospitals, and were community-dwelling persons without a history of dementia.

### Clinical measures and experimental protocol

Demographic data, including age, gender, disease duration and comorbidities [10], were recorded. The Unified Parkinson’s Disease Rating Scale (UPDRS) [11] and the Hoehn and Yahr Scale (HY) [12] were scored by a movement disorder specialist. The UPDRS sections II and III were used to describe the ability to perform ADLs and motor impairment, respectively. The HY was used to describe the severity of PD. All participants had received dopamine replace therapy and other anti-parkinsonian medications. All clinical assessments and testing were performed when the participants were in the optimal ‘on’ medication state, approximately 1 h after taking their dopaminergic medication.

Participants were classified based on their ability to perform chair rise (item 27 of the UPDRS). This item of the motor examination is a performance-based assessment. Participants were asked to arise from a straight-backed chair, with arms folded across their chest. Then, their performance was rated using the following ordinal scale: 0 = Normal; 1 = Slow, or may need more than one attempt; 2 = Pushes self-up from the arms of the seat; 3 = Tends to fall back and may have to try more than one time, but can get up without help; and 4 = Unable to arise without help [10]. Training was provided for the raters to improve intra/inter-rater reliability. All measures were performed in a clinic laboratory on the same standard-height chair for all participants. Investigators were blinded to the analysis of the results of chair rising ability, the SE-ADL and the PASE.

The Schwab and England Activities of Daily Living Scale (SE-ADL; UPDRS Section VI) was used to assess activity limitations [13]. The SE-ADL is rated from 0% (bedridden with vegetative functioning) to 100% (completely independent without awareness of difficulty) and provides the overall percentage of the ability to perform ADLs based primarily on independency and awareness of difficulty. The SE-ADL scale is an interview-based assessment.

The Physical Activity Scale for the Elderly (PASE) was used to assess physical activity [14]. The two main components of the PASE are household-related activities (e.g., housework, lawn care, home repair, gardening and volunteer activity) and structured exercises (e.g., sports, jogging, swimming, strengthening and endurance). Higher scores reflect higher levels of physical activity. The PASE represents the types of activities in which elderly persons usually participate.

The intraclass correlation coefficient of the UPDRS-ADL was 0.84 and that of the SE-ADL was 0.83 [15]. Cronbach alpha values of .85 to .92 have been reported for the UPDRS-ADL [16, 17].

### Statistical analysis

For statistical analysis, we first divided all participants into three groups based on their ability to perform chair rising (normal group, slow or > 1 attempt group, push self-up group), and we performed cross-sectional comparisons for demographic (age and sex) and clinical (years since diagnosis of PD, disease severity, motor impairment, co-morbidity, daily activity limitations, percentage of activity limitations and physical activity) characteristics among the three chair-rise groups. For the cross-sectional comparisons, we used one-way analysis of variance (ANOVA) for age and daily activity limitations (i.e., parametric variables), and Kruskal-Wallis tests for disease severity and percentage of activity limitation (i.e., non-parametric variables). For years since diagnosis of PD, motor impairment and physical activity, we applied square root transformation, then used one-way ANOVA. The ratio of males to females in each group was compared using a Chi-square test. Then, across all persons with PD, we examined if the ability to perform chair rise would be selected as a significant predictor from all measured variables for daily activity limitations, percentage of activity limitations and physical activity, using backward stepwise multivariable regression analysis (Criterion: Probability of F-to-remove ≥ .100). For all multivariable regression models identified, we confirmed non-multicollinearity among the independent variables based on variance inflation factors (VIF; all VIF < 3.0), and normal distribution of the residuals (i.e., the difference between the observed value and the predicted value in the dependent variables) using Kolmogorov-Smirnov tests.

## Results

Sixty-two (70.1%) men and 26 (29.9%) women with PD participated in the study. All participants were community-dwelling persons and received medical treatment for PD. Their mean age was 69.67 ± 8.89 years. The average time since diagnosis was 8.05 ± 5.23 years with the average HY stage was 2.47 ± 0.48. Fifty-four (61.36%) persons were able to perform a chair rise normally, 24 (27.27%) performed slowly or needed more than one attempt to rise and 10 (11.36%) had to push themselves up using the arms of the chair. Table 1 show the characteristics of the three groups (i.e., normal, slow, push self-up) in terms of demographics, motor impairment, physical activity and ADL variables. There was no significant difference in the ratio of males to females among the three groups (Table 1).

**Table 1:** Clinical characteristics of individuals with PD according to chair rise performance (*N* = 88). Figures are mean and standard deviation unless indicated otherwise

Clinical Characteristics	All (N = 88)	Normal (*N* = 54)	Slow or > 1 attempt (*N* = 24)	Push self-up (*N* = 10)	Test statistics	*P*-value (Group differences)
Age (years) ^a^	69.67 ± 8.89	68.31 ± 8.19	70.38 ± 9.35	75.30 ± 9.89	*F*(2,85) = 2.824	0.065
Gender (N and %) ^b^						
Male	62 (70.5%)	38 (70.4%)	17 (70.8%)	7 (70.0%)	*Χ*^2^(2) = 0.003	0.999
Female	26 (29.5%)	16 (29.6%)	7 (29.2%)	3 (30.0%)		
Year since diagnosis of PD ^a^	8.05 ± 5.23	8.15 ± 5.49	7.50 ± 4.87	8.85 ± 5.01	*F*(2,85) = 0.235	0.791
Disease severity (HY; Median and range) ^b^	2.5 (1.0–4.0)	2.0 (1.0–3.0)	2.5 (2.0–3.0)	3.0 (2.5–4.0)	*Χ*^2^(10) = 10.182	0.060
Motor impairment (UPDRS-Motor) ^a^	18.57 ± 8.60	15.52 ± 7.58	23.42 ± 8.15 ^**+**^	23.40 ± 7.92 ^*^	*F*(2,85) = 10.073	< 0.001
Co-morbidity (CCI) ^a^	4.77 ± 1.79	4.59 ± 1.72	4.50 ± 1.69 ^**++**^	6.40 ± 1.65 ^*^	*F*(2,85) = 4.750	0.011
Daily activity limitations (UPDRS-ADL) ^a^	12.28 ± 5.14	10.35 ± 3.79	14.58 ± 4.44 ^**+**^	17.20 ± 7.53 ^*^	*F*(2,85) = 14.057	< 0.001
Percentage of activity limitations (SE-ADL; %) ^c^	85.64 ± 9.20	87.64 ± 8.30	85.00 ± 6.90	76.67 ± 13.23 ^*^	*Χ*^2^(2) = 10.479	0.005
Physical activity (PASE) ^a^	100.50 ± 63.13	112.90 ± 61.40	95.21 ± 60.27 ^**++**^	46.18 ± 52.64 ^*^	*F*(2,85) = 7.548	0.001

^a^ One-way ANOVA; ^b^ Chi-square test; ^c^ Kruskal-Wallis test

Significant pair-wise differences (*p* < .05): * Normal vs. Push self-up; ^**+**^ Normal vs. Slow; ^**++**^ Slow vs. Push self-up

Note: *ADL* Activities of Daily Living, *CCI* Charlson Co-morbidity Index, *HY* Hoehn and Yahr Scale, *PASE* Physical Activity Scale for the Elderly, *PD* Parkinson’s disease, *SE-ADL* Schwab and England Activities of Daily Living Scale, *UPDRS* Unified Parkinson’s Disease Rating Scale

Persons who pushed themselves up had more severe PD, more severe motor impairment, higher co-morbidity, more ADL limitations and lower physical activity than persons who could rise from a chair normally. Persons who rose from a chair slowly or needed more than one attempt had more severe motor impairment and more daily activity limitations than those who performed normally. Persons who pushed themselves up had more co-morbidities and lower SE-ADL scores than those who rose from a chair slowly.

Variables of interest that were significantly different among the chair rise groups (UPDRS-ADL, SE-ADL and PASE) and demographics (age, sex, disease duration, disease severity, co-morbidity, motor impairment and chair rise groups [normal, slow and push self-up]) were entered as independent variables in the initial backward stepwise multivariable regression model. The model identified sex, motor impairment and the slow and push self-up group as significant predictors for UPDRS-ADL and HY; identified the push self-up group as a significant predictor for SE-ADL; and identified age and the push self-up group as significant predictors for PASE (Table 2). The total variance (i.e., adjusted R^2^) was 0.374, 0.239 and 0.142 for UPDRS-ADL, SE-ADL and PASE, respectively (all models *p* < .05).

**Table 2:** Backward stepwise regression analysis assessing the variance in UPDRS-ADL, SE-ADL and PASE explained by chair-rise ability after accounting for physical impairments

Dependent Variables	Independent Variables	*β* (*p*-value)	R^2^	Adjusted R^2^	F of model (*p*-value)
UPDRS-ADL (resulting model)	Sex	.224 (.011)	.403	.374	*F*(4, 83) = 13.989 (<.001)
	UPDRS-Motor	.309 (.001)			
	Slow	.253 (.008)			
	Push self-up	.357 (<.001)			
SE-ADL (resulting model)	HY	−.352 (.001)	.257	.239	*F*(2,83) = 14.344 (<.001)
	Push self-up	−.266 (.009)			
PASE (resulting model)	Age	−.264 (.011)	.162	.142	*F*(2,85) = 8.208 (.001)
	Push self-up	−.250 (.016)			

Note: *UPDRS* Unified Parkinson’s Disease Rating Scale, *SE-ADL* Schwab and England Activities of Daily Living Scale, *PASE* Physical Activity Scale for the Elderly, *HY* Hoehn and Yahr Scale

## Discussion

Our results revealed, for the first time, the association of chair rise ability, ADL and physical activity in community-dwelling persons with PD. As we hypothesized, impaired ability to perform the chair rise was found to be associated with decreased ability to perform ADLs. Poor chair rise ability (i.e., pushing self-up) was a significant predictor for limited ADLs and physical inactivity. Our finding is in agreement with a previous study that performance of the 5-time sit-to-stand test was moderately correlated with physical activity in those with PD [18]. Impaired ability to perform chair rise likely leads to impaired ability to perform the 5-time sit-to-stand test. We found that persons with PD who demonstrated poorer ability to perform chair rise had more severe disease severity, more motor impairment, more comorbidity, lower UPDRS-ADL scores, lower scores on SE-ADL and lower level of physical activity than persons who performed better on the chair rise task. The findings suggested that the chair rise task is a skill that can help determine the functional level of persons with PD. Persons with more severe PD perform the chair rise more slowly than those with less severe PD. The common features of PD, including postural instability, bradykinesia and less strength in the extremities, can contribute to impaired chair rise performance. A study by Pääsuke et al. reported a longer chair-rise time and lower maximal rate of vertical-ground-reaction-force development while rising from a chair in PD patients, compared to those of controls [1]. Greater hip strength was also found to be related to better sit-to-stand ability in subjects with PD. Reduced strength, particularly at the hip, may be one factor that contributes to the difficulty of persons with PD in rising from a chair [1].

Persons with PD have strength deficits that cause functional difficulties [5]; for example, 78% of those with PD have trouble walking and 81% have difficulty rising from a chair [2]. Repeated sit-to-stand tests are often used as a clinical measure of lower extremity strength [19]. Persons with PD produce reduced extension forces at the hip compared to control subjects, which can contribute to difficulty rising from a chair [1]. Older adults require knee extensor relative muscular effort of approximately 80% of their strength reserves when ascending from a chair [20]. Impaired balance and bradykinesia can also affect the ability to rise from a chair [19, 21]. The additional physical demands of getting up from a chair can prevent persons with PD from performing functional activities and eventually cause them to more dependent in ADLs.

In persons with PD, three strategies to rise from a chair — including slow rise, multiple attempts to rise and using arms to rise — may reflect a reduced ability of the lower extremities to generate sufficient extensor force [1] to lift the buttocks from the chair. The slow rise or multiple attempts to rise strategies (without using arms) may also indicate a reduced ability to perform ADLs that is too small to be retained in the models for overall ability to perform ADLs and overall physical activity (Table 2). The need to use arms to push while rising up from a chair might represent a potential threshold that marks the beginning of sedentary behavior and decreasing ADL independence in persons with PD. However, the threshold of becoming sedentary or losing ADL independence has not been studied extensively. This assumption of sedentary threshold requires further investigation.

In addition, our findings may reflect the bidirectional relationship between physical activity levels and physical fitness. The decreased physical fitness from having PD can lead to reduced physical activity and less ability to perform ADLs; chair rising ability may merely be an associated symptom of such changes. Since correlation studies do not infer causation, further research is required to evaluate whether improving the ability to perform the chair rise will lead to improved ADL function and increased physical activity.

The clinical relevance of these findings is that, by administering the chair rise test, a clinician can possibly infer the ADLs, activity limitation and physical activity of the patient and gauge the level of independence of that patient. This knowledge is important for a clinical provider to prepare for proper management (e.g., rehabilitation referral, social support referral, recommend exercise intervention and ambulatory device consideration). Proper management may prevent or delay a deterioration in the ADLs and physical activity of the patient.

Future research should examine chair rising abilities over time, to monitor how the performance changes contribute to ADL limitations in persons with PD. An effective intervention focusing on the chair rise performance can be developed to delay the decline in ADL performance and prevent functional dependence in persons with PD.

### Limitations of the study

There are some limitations of the study which must be addressed. First, deficits in cognitive function are common in PD, and might influence the chair rising ability. However, this parameter was not investigated in this study and needs further investigation. Second, we did not study the relationship of leg strength with chair rise ability in relation to physical activity levels. Third, the relative added information provided by the chair rise test apart from disease metrics is unknown. In addition, we did not investigate sub-classification of our participants. Investigating the association between chair rise ability and physical activity in persons with motor subtypes of PD like tremor dominant and postural instability-gait disturbance is recommended. Also, the number of participants in the push self-up group was relatively small (*n* = 10). Although we found significant differences even with this small sample size, caution should be taken when interpreting the results, and results from a larger study are recommended to confirm the generalizability of our results.

### Implication for practice

The test used for chair-rising ability is a subset of the UPDRS-motor scale. A reduced ability in chair-rising would therefore inevitably cause a reduced score on the UPDRS motor scale. Clinicians might be able to postulate that persons who perform poorly on the chair-rising task might have limited functional ability and reduced ADL performance. This information can be helpful in providing proper clinical management.

The relevance in clinical practice would be increased by discussion of cut-off points or via the discussion of minimal abilities for varying levels of independence.

## Conclusions

Ability to rise from a chair was associated with ADL limitation and physical activity in persons with PD. Poor ability to rise from a chair may prevent persons with PD from living independently and engaging in physical activity. The chair rise test is a quick, easily administered measure that can be useful to grossly evaluate the ability to perform ADLs and the level of independence in persons with PD.

## Data Availability

The datasets used and/or analyzed during the current study are available from the corresponding author on reasonable request.

## Abbreviations

PD: Parkinson’s disease
ADL: Activities of daily living
UPDRS: Unified Parkinson’s Disease Rating Scale
HY: Hoehn and Yahr Scale
SE-ADL: Schwab and England Activities of Daily Living Scale
PASE: Physical Activity Scale for the Elderly
CCI: Charlson Co-morbidity Index
